# Quality of registration of antenatal, intrapartum, and newborn information in the Georgian birth registry

**DOI:** 10.1186/s13690-024-01479-y

**Published:** 2024-12-24

**Authors:** Charlotta Rylander, Tinatin Manjavidze, Ingvild Hersoug Nedberg, Maia Kerselidze, Erik Eik Anda

**Affiliations:** 1 Department of Community Medicine, UiT The Arctic University of Norway, Tromsø, 9037 Norway; 2 Department of Medical Statistics, National Center for Disease Control and Public Health, Tbilisi, Georgia; 3 Department of Health and Care Sciences, UiT The Arctic University of Norway, Tromsø, 9037 Norway

**Keywords:** Birth registry, Health registry, Data quality, Maternal health, Pregnancy, Childbirth

## Abstract

**Background:**

The Georgian Birth Registry (GBR) is a comprehensive digital birth registry covering 99.8% of births nationwide. By law, registration in the GBR is mandatory, with data primarily transferred from medical records (MRs) by designated personnel at medical facilities. We aimed to assess the correspondence of the registration of selected variables between GBR and MRs.

**Methods:**

We randomly selected 1,044 women who gave birth in 2018. Data were extracted from the GBR on 27 variables related to pregnancy, childbirth, and the newborn and individually linked to the MRs. We specifically compared the agreement of dichotomous, ordinal, and date variables between the GBR and the MRs to assess the consistency of individual registrations.

**Results:**

Of the 27 dichotomous, ordinal, and date variables, 22 displayed more than 95% complete agreement with the information in the MRs. The prevalence of maternal morbidity registered in the MRs was lower than expected, while the proportion of fetuses with transverse lies was higher than expected.

**Conclusions:**

Most antenatal, intrapartum, and newborn information registered in the GBR has satisfactory agreement with the MRs, with error typical for single data entry system. The lower-than-expected prevalence of gestational diabetes, preeclampsia, hypertensive disorders, and postpartum hemorrhage registered in the MRs, as well as the higher-than-expected prevalence of transverse fetal presentation, warrants in-depth investigation to ensure that the quality of care is satisfactory and to further improve registration in both the MRs and GBR. Therefore, our findings indicate that while the agreement between the GBR and MRs is generally high, MRs are sometimes incomplete or incorrect for certain conditions.

**Table:** 

**Text box 1. Contributions to the literature**
• The Georgian Birth Registry (GBR) is a nationwide digital health registry in the Republic of Georgia. Validation of the quality of registrations in the GBR has not been performed.
• Most information in the GBR related to pregnancy care, childbirth and the newborn was in satisfactory agreement with information in the corresponding medical records (MRs).
• The registered occurrences of certain diseases during pregnancy and complications during childbirth were lower or higher than the same conditions in other countries.
• An increased focus of incomplete or incorrect registrations of certain conditions in the MRs and their associated effects is warranted.

## Background

Georgia is an upper-middle-income country in the Caucasus region. Over the past 15 years, Georgia has made considerable progress in maternal and neonatal healthcare. Through the universal health coverage (UHC) system implemented in 2013, both antenatal care (ANC) and childbirth in hospitals are covered by the government. Hence, most women (99.8% in 2021) give birth in a birth facility and have at least one ANC visit during pregnancy (94.4%) [1, 2]. Private healthcare providers predominate and although ANC and childbirths at private clinics are covered by the UHC, additional services are offered for extra fees. The doctor-to-nurse ratio in Georgia is high, and childbirths are primarily managed by obstetricians, as midwifery is not established as the primary model of care. Georgia has reached the millennium development goal for under-5 child mortality, but the neonatal mortality rate (5.6 per 1,000 live births in 2021) remains among the highest in Europe and the proportion of cesarean section (CS) births in Georgia is among the highest worldwide (44.7% in 2021) [1]. For comparison, the neonatal mortality rate varied between 0.5 and 4.3/1000 live births in Europe in 2019 and the proportion of CS births was 21.1%, globally in 2010–2018 [3, 4].

In 2016, the national digital Georgian Birth Registry (GBR) was implemented [5]. The main aim of the GBR is to replace aggregated monthly data with continuous digital individual-level data. The GBR aimed to improve the availability, accessibility, and applicability of information for care during pregnancy and from one pregnancy to the next and to improve the continuity of information between ANC providers and hospitals. The GBR includes information on ANC, childbirth, and hospital stay for the mother and newborn. It covered 99.8% of all births in Georgia in 2021 [6]. Registration of core information such as maternal age, complications during pregnancy, laboratory results, gestational age, fetal presentation, onset of labor, type of birth, complications during childbirth, newborn characteristics, and newborn diseases is mandatory for ANC providers and hospitals. A physician or nurse inputs the information into the GBR either continuously during a doctor’s consultation or extracts the information from medical records (MRs) to enter it into the GBR at a later stage. Some clinics use a combination of these methods. Other health facilities employed non-medical personnel to extract information from MRs and input it into the GBR. Each user has its own user ID; currently, there are approximately 2,500 GBR users, of which approximately 1,700 have a medical background. Hence, GBR is a single-entry (information entered by one person) system that includes many programmed edit checks to avoid human mistakes and improve data quality. For instance, implausible values are flagged and mandatory information must be filled in to close and submit the file. The GBR is currently used as the primary source of maternal and newborn data for national statistics and birth registry research.

All the data, whether paper-based or electronic, contain errors. However, there is a limited number of publications on data quality and proportions of errors when transferring data from one source to another, although such information holds significant importance for others trying to improve data quality and for those who must consider errors when analyzing data. A preprint of a systematic review from 2023, including 93 papers concerning data transfer, reported error proportions of 2 − 2,784 per 10,000 entry fields, or approximately 0.02–28% [7]. For single-entry databases, the error proportions were 0.04-6.5%, which were similar to those of optical scanning.

To further improve GBR and add to the pool of literature concerning the proportion of errors in data processing methods, a quality study of core information is warranted. Hence, the aims of this study were twofold: (i) to individually link records from the GBR with the corresponding MRs to compare the quality of the registration of selected antenatal, intrapartum, and newborn information across the two data sources (ii) to compare the registered prevalence of complications during pregnancy and childbirth in the MRs with corresponding information from other countries.

## Methods

Of the 50,464 women registered in the GBR who gave birth in 2018, we randomly selected 1,044 women and their newborns (*n* = 1,049). The National Center for Disease Control and Public Health in Georgia (NCDC) contacted the hospitals where the women gave birth and requested a copy of their MRs. MRs were also collected from ANC clinics; if a woman attended more than one ANC clinic, MRs were collected from all attended clinics.

For each woman, the NCDC extracted individual information from the GBR and linked it with the corresponding data from the MRs. Data from the GBR was automatically extracted, while the data from the MRs was manually extracted using a double-entry method to ensure accuracy. A predefined protocol and spreadsheet facilitated systematic data collection. One research assistant reviewed the MRs, and another entered the information into the spreadsheet. Subsequently, they double-checked the entered data to ensure consistency and accuracy. The variables covered (i) ANC-related information (parity, number of previous CS, number of previous spontaneous abortions, and complications during pregnancy), (ii) intrapartum information (date of birth, date of discharge, death or transfer, presentation of fetus, onset of labor, indication of CS, complications during birth and vital status [liveborn or stillborn]), and (iii) newborn information (gestational age [GA] week at birth, newborn birth weight, fetal heartbeat measured by cardiotocography [CTG] at admission, main diagnosis of newborn morbidity, and transfer to the neonatal intensive care unit [NICU]). Complications during pregnancy and childbirth and newborn morbidity were registered using the International Classification of Diseases version 10 (ICD10) codes. We selected the most prevalent conditions: gestational diabetes (ICD10:O24), preeclampsia (ICD10: O14), and hypertensive disorders (ICD10: O11, O13, O14, O15, O16) were included for complications during pregnancy; for complications during childbirth, we included postpartum hemorrhage (PPH, ICD10:O72). For newborn morbidity, we included respiratory distress (ICD10: P21), infection (ICD10: P35-P39), and congenital malformation (ICD10: Q00-Q99). If the abovementioned ICD10 codes were not registered, the condition was considered absent. Therefore, 27 variables were included in this study.

Ordinal and date variables are presented as the proportion of observations with complete agreement (identical entries in both sources) between GBR and MRs, the proportion of deviating registrations +/- one category, and the proportion of observations deviating by more than one category. To quantify complete agreement, we compared data for each variable and each woman in the GBR against the corresponding entries in the MRs. We then calculated the proportion of women whose information matched exactly across both sources, dividing the number of women with identical records by the total number of women (*n* = 1,044). For newborn birthweight (*n* = 1,049), we defined deviating observations as differences in birthweight of +/- 100 g between the GBR and MRs. Missing observations in the GBR and MRs were also reported. The quality of dichotomous variables was assessed by calculating the frequencies and proportion of complete agreement between the GBR and MRs, as well as the frequencies and proportions of variables registered in only the GBR or MRs. We also calculated the prevalence of conditions with 95% confidence intervals (CIs) registered in the MRs. All statistical analyses were performed using the statistical software Stata, version 17.0 (StataCorp, 4905 Lakeway Drive, College Station, TX, USA).

## Results

Of the 1,044 included women, 384 (37%) were pregnant with their first child, and five women were pregnant with multiples. The median GA week was 39, and 39.1% had a CS birth.

In the GBR, between 92.4 and 99.1% of individual records regarding parity, number of previous CSs, date of birth, date of death, transfer or discharge, and GA week at birth were in complete agreement with the corresponding entries in the MRs. However, information about previous spontaneous abortions and number of ANC visits displayed lower proportions of complete agreement with the MRs, at 81.8% and 73.2%, respectively (Table 1). Disagreeing registrations regarding parity and ANC visits mainly deviated by one category, while deviating registrations of previous CS mainly disagreed with more than one category. Specifically, for ANC visits, 69.5% of disagreeing registrations were input with a higher number in the GBR compared with the corresponding MRs. The number of missing registrations of parity; number of ANC visits; date of birth; date of discharge, death, or transfer; and newborn birthweight in the GBR ranged from 0 to 7.5%, and the proportions were comparable to missing information in the MRs. There was a higher proportion of missing values in the GBR for number of previous CS (11.5% vs. 8.8%) and number of previous spontaneous abortions (23.9% vs. 12.4%) compared to the MRs. However, for GA week at birth, there were zero missing values in the GBR, while 5.0% of registrations in the MRs were impossible to interpret because of registration mistakes. Of 1,049 newborns, 98.3% had identical birthweight recorded in both the GBR and the MR; 0.6% had recorded birthweight deviation of less than or equal to +/-100 g, and 1.1% of newborns were registered with a larger discrepancy in birthweight between the two data sources.

**Table 1:** Comparison of registration of ordinal, date, and continuous antenatal, intrapartum, and newborn variables between the Georgian Birth Registry (GBR) and medical records (MRs) in 2018, n women = 1,044 and n newborn = 1,049

	Complete agreement (%)	Deviating registrations (%)		Missing in the MR, *n* (%)	Missing in the GBR, *n* (%)
		+/- 1 category	> 1 category		
**Antenatal variables**					
Parity	95.8	58.1	41.9	90 (8.6)	79 (7.6)
Number of CS	92.3	23.8	76.2	92 (8.8)	120 (11.5)
Number of spontaneous abortions	81.8	NA	NA	129 (12.4)	250 (23.9)
Total number of ANC visits	73.2	73.2	26.8	80 (7.7)	79 (7.6)
**Intrapartum variables**					
Date of birth	99.1	44.4	55.6	0 (0)	0 (0)
Date of discharge, death or transfer of the mother	98.9	33.3	67.7	0 (0)	0 (0)
**Newborn variables**					
GA week at birth	93.7	NA^a^	NA^a^	52 (5.0)^b^	0 (0)
Newborn birthweight^c^	98.3	0.6	1.1	0 (0)	0 (0)

^a^Due to large differences in the proportions of missing values in the MRs and GBR, the proportion of misclassified observations +/- 1 or > 1 category was not computed for GA week at birth and the number of spontaneous abortions. ^b^52 observations on GA week at birth were coded as 38/39 in the medical records. This type of coding is impossible in GBR because of programmed edit checks, which make it impossible to compare the agreement between GBR and MRs for these observations. ^C^Deviations of +/- 1 category of birthweight correspond to +/- 100 g. Deviations of more than 1 category of birthweight correspond to deviations of more than 100 g between the two data sources.

More than 99% of the registrations of gestational diabetes, preeclampsia, and hypertensive disorders in the GBR were in complete agreement with the corresponding MRs (Table 2). According to the MRs, 0.2% of the pregnant women had gestational diabetes, 0.3% had preeclampsia, and 0.6% had hypertensive disorders. Furthermore, more than 98% of registrations in the GBR related to fetal presentation and onset of labor were in complete agreement with the MRs. According to the MRs, 93.9% of fetuses had cephalic presentation, 1.5% had transverse lie, and 3.9% had breech presentation. Approximately 88% of births had a spontaneous onset of labor, and 10.5% had planned CS. Of the total number of CSs, 28.4% reported previous CS as the main indication for the present CS, 23.5% reported maternal request as the indication for CS, and 2.2% reported a transverse lie of the fetus. Note that the proportions did not sum up to 100%, as not all indications for CS were considered in the present study.

**Table 2:** Comparison of registration of dichotomous antenatal, intrapartum, and newborn variables between the Georgian Birth Registry (GBR) and medical records (MRs) in 2018, n women = 1,044 and n newborn = 1,049

	Complete agreement: *n* (%)	Registered as present in the MR but not in the GBR: *n* (%)	Registered as present in the GBR but not in the MR: *n* (%)	Registered prevalence (%) in MR (95% CI)
**Antenatal variables**				
Gestational diabetes	1,040 (99.6)	2 (0.2)	2 (0.2)	0.2 (0.0–0.7)
Preeclampsia	1,043(99.9)	1 (0.1)	0 (0.0)	0.3 (0.1–0.8)
Hypertensive disorders	1,040 (99.6)	6 (0.4)	0 (0.0)	0.6 (0.2–1.2)
**Intrapartum variables**				
Presentation of the fetus: cephalic	1,035 (99.1)	7 (0.7)	2 (0.2)	93.9 (92.2–95.2)
Presentation of the fetus: transverse	1,042 (99.8)	2 (0.2)	0 (0.0)	1.5 (0.9–2.5)
Presentation of fetus: breech	1,035 (99.1)	3 (0.3)	6 (0.6)	3.9 (2.8–5.3)
Onset of labor: spontaneous	1,025 (98.2)	3 (0.3)	15 (1.4)	87.5 (85.3–89.4)
Onset of labor: planned CS	1,029 (98.6)	12 (1.1)	2 (0.2)	10.5 (8.7–12.5)
Onset of labor: induction of labor	1,039 (99.5)	3 (0.3)	1 (0.1)	2.1 (1.3–3.2)
Indication for CS: previous CS*	399 (97.8)	7 (1.7)	2 (0.5)	28.4 (24.1–33.1)
Indication for CS: maternal request*	394 (96.6)	13 (3.2)	1 (0.2)	23.5 (19.5–28.0)
Indication for CS: transverse lie*	406 (99.5)	1(0.2)	1 (0.2)	2.2 (1.0–4.1)
Postpartum hemorrhage	1,043 (99.9)	0 (0.0)	1 (0.1)	0.9 (0.4–1.6)
**Newborn variables**				
Fetal heartbeat measured by CTG on admission (yes/no)	949 (90.5)	99 (9.4)	1 (0.1)	99.5 (98.9–99.8)
Main neonatal diagnosis: respiratory distress	1,040 (99.1)	7 (0.7)	2 (0.2)	5.3 (4.1–6.9)
Main neonatal diagnosis: infection, unspecified	1,041 (99.2)	5 (0.5)	3 (0.3)	1.8 (1.1–2.8)
Main neonatal diagnosis: congenital malformation	1,048 (99.9)	1 (0.1)	0 (0.0)	1.2 (0.7–2.1)
NICU admission	1,041 (99.2)	8 (0.8)	0 (0.0)	7.4 (5.9–9.2)
Vital status (liveborn/stillborn)	1,049 (100)	0 (0.0)	0 (0.0)	0.8 (0.3–1.5)

*Numbers relate to total number of CS. Abbreviations: CS: cesarean section; CTG: cardiotocography; NICU: neonatal intensive care unit

In the GBR, 99.9% of individual records of PPH were in complete agreement with the corresponding entries in the MRs, and the registered prevalence according to the MRs was 0.9%. For fetal heartbeats measured by CTG upon hospital admission, 90.5% of registrations in the GBR were in complete agreement with the MRs. According to the MRs, 99.5% of fetuses had confirmed heartbeats monitored by CTG upon admission to the hospital for childbirth; however, 9.4% of fetuses with confirmed heartbeats measured by CTG and registered in the MRs, were not registered as such in the GBR.

We found high complete agreement (> 99%) between registrations of respiratory distress in newborns, newborn infections, congenital malformations, and NICU admission in the GBR and MRs, and 100% agreement for vital status. According to the MRs, the incidence of respiratory distress in newborns was 5.3%, 1.8% of newborns had an infection, and 1.2% were born with a congenital malformation. 7% of the newborns were admitted to the NICU after birth, and 0.8% were stillborn (Table 2).

## Discussion

The GBR is a unique data source that contains information collected during ANC, childbirth, and the postpartum period for almost all births in Georgia. The results of the present study confirmed that most of the selected antenatal, intrapartum, and newborn variables registered in the GBR were in reasonable agreement with the corresponding information entered in the MRs. The exceptions were the number of previous spontaneous abortions, ANC visits, GA week at birth, and fetal heartbeats measured by CTG upon hospital admission. It is important to keep in mind that this study evaluates the transfer of data from MRs to a digital registry (the GBR), and not errors in the MRs themselves. In this study, the proportion or registration errors was mainly below 5%, which is comparable to other studies using single-entry data processing methods [7]. The data used in this study was collected during the third year following the implementation of the nationwide GBR. Some omissions may be attributed to healthcare providers’ lack of familiarity with the system, as adapting to, and fully mastering a new electronic system, often requires a significant amount of time.

### Antenatal variables

For the selected ANC-related information, the individual records for parity and the number of previous CSs in the GBR showed complete agreement with the corresponding entries in the MRs at proportions of 96% and 92%, respectively. There was a larger proportion of missing information regarding previous CSs in the GBR than in the MRs, which explains some of the disagreement between the registrations. Likewise, the number of previous spontaneous abortions had almost twice as many missing values in the GBR as in the MRs, which contributed to a lower proportion of registrations with complete agreement (82%). Interestingly, if all entries with missing information in either MRs or GBRs was excluded, the proportion of complete agreement would increase to 96.3% for previous spontaneous abortions. In 2018, it was not mandatory to register the number of previous spontaneous abortions in the GBR, which explains the larger number of missing values in the GBR entries. However, since 2019, registration has become mandatory; hence, the number of missing values in GBR has decreased considerably. In 2019, the registration of previous CS became mandatory in the GBR. Disagreements in the registration of the number of ANC visits between the GBR and MRs are explained by changes in the reimbursement process related to ANC visits. In Georgia, costs related to ANC are reimbursed by the Social Service Agency, and after the implementation of the GBR, the reimbursement process was conducted electronically. In the present study, only 73% of the individual records regarding number of ANC visits were in complete agreement with the corresponding entries in the MRs. Of disagreeing entries, 70% were recorded with a higher number in the GBR than in the MR. Thus, because the GBR is currently the primary data source for reimbursement, it is highly likely that the registration of ANC visits is more complete in the GBR than in the MRs.

Although we demonstrated excellent agreement between the registered information on maternal morbidity in the GBR and the corresponding information in the MRs, our results also highlight that the prevalence of gestational diabetes, preeclampsia, and hypertensive disorders registered in the MRs was low. Only 0.2% of pregnant women in Georgia were registered with gestational diabetes, whereas recent systematic reviews and meta-analyses estimated its prevalence in Europe and Asia to be approximately 11% [8, 9]. Large regional variations in prevalence have been reported; for example, the pooled prevalence in Northern Europe in 2014–2019 was approximately 9% [8], 31.5% in Eastern Europe [8], 1.5% in Nepal [9], and 22.9% in Saudi Arabia [9]. These large differences can be partly explained by the various registration regimes, diagnostic criteria, and testing strategies, as there is no universal consensus. Nevertheless, a prevalence of less than 1% in Georgia is highly unlikely and suggests that either pregnant women remain undiagnosed, which is unfortunate since gestational diabetes increases the risk of CS birth, macrosomia, preterm birth, low 1-minute Apgar score, and born large for gestational age [10], or that the disease is poorly registered in the MRs. Likewise, although there are disagreements worldwide regarding the classification and diagnosis of preeclampsia and hypertensive disorders in pregnancy [11], the prevalence of these conditions registered in the MRs in Georgia (preeclampsia:0.3% and hypertensive disorders:0.6%) was considerably lower than the crude global prevalence of preeclampsia of 2.3% in 2002–2010 [12], although regional variations exist, e.g., 4.2% in the western Pacific region and 1.2% in the eastern Mediterranean region. Hypertensive disorders are usually present in 5.2–8.2% of all pregnancies [13], clearly highlighting that the prevalence registered in the MRs in Georgia is suspiciously low. Because hypertensive disorders during pregnancy are a major cause of maternal and newborn morbidity and mortality [14, 15], it is crucial that women are correctly diagnosed during ANC and receive optimal treatment. Hence, Georgian stakeholders should identify whether the low prevalence of preeclampsia, hypertensive disorder and gestational diabetes in MRs is due to a lack of diagnosis and, accordingly, proper management or if it is solely a registration problem.

### Intrapartum variables

More than 96.6% of the individual records of the selected intrapartum-related information in the GBR were in complete agreement with the corresponding entries in the MRs, which can be considered sufficient. For instance, 99.1% of birth dates were identical in the GBR and MRs, and disagreeing registrations can be explained by registration mistakes in either the GBR or MRs. Registration of fetal presentation in the GBR had excellent agreement with MRs; however, according to the MRs, approximately 4.0% of fetuses had a breech presentation and 1.5% had a transverse lie. The prevalence of breech presentation varies with gestational age [16] and complicates approximately 3–5% of pregnancies, which is in line with the registrations in MRs [17]. In contrast, transverse lie is a very rare condition that affects less than 0.5% of term pregnancies [17]. In a study of 11,957 singleton births over a 10-year period in Finland, a transverse lie was present in 0.12% of births [18]. Based on these numbers, the prevalence of 1.5% of transverse lies according to the MRs seems high. Transverse lie is an absolute indication for CS birth, and Georgia has among the highest CS rates in the world, reaching 44.7% in 2021 [6]. The emergency CS rate in Georgia is also unnaturally high, which could indicate intentional misclassification of planned CS as an emergency CS [19]. The clinical guidelines in Georgia state that CS should only be performed upon medical indication and that obstetricians are not encouraged to perform CS upon maternal request without a medical indication [20]. Therefore, it is surprising that in 24% of the CS births, maternal requests were registered in the MRs as an indication for CS, clearly suggesting that the clinical guidelines were not entirely followed. The healthcare system in Georgia is privatized, and CS births receive a higher monetary reimbursement from the state than vaginal births, which could be a driver of the high CS rates in the country [21]. In the present study, we also found that 2.2% of CS births had transverse lies as an indication for CS registered in the MRs. This number also appears unnaturally high and may be due to misclassification; however, validation of the MRs was outside the scope of this study.

Although the individual registrations of PPH in the GBR demonstrated close to 100% agreement with corresponding entries in the MRs, the registered prevalence in the MRs was only 0.9%. PPH within 24 h of childbirth occurs in 1.2–12.5% of births and is a leading cause of maternal morbidity and mortality; hemorrhage after 24 h is much less common and occurs in < 1% of births [22]. In Norway, almost 32% of women giving birth in 2021 experienced blood loss of 500 mL or more, whereas 4.5% of mothers lost more than 1500 mL and required blood transfusion [23]. In general, misdiagnosis of PPH is common, as indicated by large variations in prevalence, mainly because of the underestimation of blood loss, lack of proper clinical protocols, and lack of education and training for medical personnel [24]. The low registered prevalence in Georgia is a concern, especially because hemorrhage was identified as the leading direct cause of maternal deaths in Georgia from 2014 to 2017 [25]. Hence, it is important to identify the reason for the low reported prevalence of PPH and assess whether clinical practices regarding the diagnosis and treatment of PPH are adequate.

### Newborn variables

Of the selected newborn variables, all except fetal heartbeats measured by CTG upon hospital admission displayed reasonable agreement between individual registrations in the GBR and corresponding entries in the MRs. GA weeks at birth were in complete agreement with the MRs in approximately 94% of the registrations. There were 54 women who had two different GA weeks registered in the MRs. This is impossible in the GBR because of programmed edit checks. Because of these reporting mistakes, these registrations were coded as missing when extracting data from the MRs, which reduced the proportion of observations with complete agreement between the GBR and MRs. There were no substantial differences in newborn birthweights registered in the two data sources, which was reassuring. Almost 10% of women lacked information about fetal heartbeats monitored by CTG upon hospital admission in the GBR, whereas the information was present in the MRs. Speculating about the reason for the lack of registration of this variable in the GBR is challenging. The variable is described as “monitoring of fetal heartbeat with CTG upon admission to the hospital,” which is quite specific. It is possible that due to time constraints, heartbeat monitoring was not performed immediately upon admission but was conducted later, hence it was recorded in the MRs but not in the GBR. Additionally, the heartbeat might have been monitored using intermittent auscultation, which may not have been recorded in the GBR due to the specific wording of the question. In either case, our results show that variables related to the registration of fetal heartbeats upon hospital admission should be used and interpreted with caution.

More than 99% of the individual registrations in the GBR regarding newborn diagnoses such as respiratory distress, newborn infection, and congenital malformations were in complete agreement with corresponding entries in the MRs. Based on the MRs, 5.3% of newborns were diagnosed with respiratory distress, 1.8% had infections, and 1.2% had congenital malformations. Worldwide, approximately 7% of newborns are diagnosed with respiratory distress [26]; however, the incidence varies across countries and is higher among premature children [27]. Additionally, children born via CS usually have a higher incidence of this condition [28]. In Norway, the incidence of respiratory distress was approximately 1.6% in 2021 [23]. Given that both the proportion of CS and the incidence of early neonatal deaths (the main cause of death being prematurity) in Georgia are considerably higher than those in Norway, an incidence of 5.3% could be reasonable. It is challenging to compare the number of newborn infections with other studies, as it is not expected to be similar across countries, owing to variations in resources and clinical practices. Additionally, infections diagnosed after transfer to the NICU at another hospital may not be registered in the GBR. Therefore, this variable should be interpreted with caution.

The incidence of 1.2% of congenital malformations registered in the MRs is considerably lower than that in Sweden in 2016 (3.2% of liveborns) [29] and in Norway in 2021 (4.0%) [23]. However, we did not expect a comparable incidence, as the MRs and GBR only covered the period from birth until discharge from the hospital, while both Norway and Sweden had a longer registration period and included malformations from abortions performed after 12 weeks of gestation. This is a limitation of the data in the GBR and, in fact, of many birth registries. Unless data on congenital malformations are complete from early pregnancy until a certain time after birth (e.g., 1 year), these data should be used with caution.

The registration of NICU admissions in the GBR displayed close to 100% agreement with the MRs (99.2%), which was reassuring. NICU admission registrations in the GBR have previously been validated by crosschecking registrations with a hospitalization registry in Georgia [30]. During that study, we found that only 0.39% of NICU transfers were not registered in the GBR, which was a very small proportion. Hence, information regarding NICU admissions in the GBR can be considered of high quality. Likewise, newborn vital status, displayed 100% completed agreement between the GBR and MRs.

### Strengths and limitations

This study assessed the quality of individual registrations of selected core variables in the GBR compared with the corresponding entries in the MRs. The random selection of participants ensured that the results reflected the quality of registration across all types of health facilities in Georgia. However, our study did not validate the correctness of information on MRs. For such a study, a third source of information is needed. Because of the COVID-19 pandemic, the extraction of data for this study was delayed; since 2018, the GBR has implemented more programmed edit checks and made additional core variables mandatory. Therefore, the current quality of registration for several core variables in the GBR is likely higher than that presented in this study. Lastly, the research assistants responsible for data extraction were not maternity care professionals, which may have affected the accuracy of certain data points. However, all data was extracted according to a double-entry procedure to minimize extraction mistakes.

### Implication of findings

Our findings indicate that although most individual records in the GBR largely align with the corresponding entries in the MRs, there are notable instances with incomplete or incorrect registrations for certain conditions in the MRs. Healthcare authorities should therefore evaluate whether the lower-than-expected prevalence of maternal diseases during pregnancy and PPH is due to underdiagnosis of critical conditions, leading to missed treatment opportunities, or if it stems from poor registration practices. Similarly, the unexpectedly high prevalence of transverse fetal presentation, along with the high percentage of CS births, should be thoroughly investigated.

## Conclusion

Most antenatal, intrapartum, and newborn information registered in the GBR is in satisfactory agreement with the information registered in the MRs. The lower- or higher-than-expected prevalence of maternal diseases during pregnancy, PPH, and transverse fetal presentation registered in the MRs warrant in-depth investigation to ensure that clinical guidelines are followed, pregnant women receive optimal care, and to further improve registration in both the MRs and GBR. Although most variables displayed good agreement between the GBR and the MRs, our findings suggest that the MRs are incomplete or incorrect for certain conditions.

## Data Availability

The datasets used and/or analyzed in the current study are available from the National Center for Disease Control and Public Health of Georgia (info@ncdc.ge) upon reasonable request.

## Abbreviations

ANC: Antenatal care
CS: Cesarean section
CTG: Cardiotocography
COVID-19: Coronavirus disease
GA: Gestational age
GBR: Georgian Birth Registry
ICD10: International Classification of Diseases version 10
MR: Medical record
NCDC: National Center for Disease Control and Public Health in Georgia
NICU: Neonatal intensive care unit
PPH: Postpartum hemorrhage
UHC: Universal health coverage
